# Diffuse Large B-cell Lymphoma Presenting as a Cardiac Mass

**DOI:** 10.7759/cureus.59755

**Published:** 2024-05-06

**Authors:** Abdullah Nasir, Yash B Patel

**Affiliations:** 1 Internal Medicine, Trinity Health Ann Arbor Hospital, Ann Arbor, USA

**Keywords:** right atrial mass, chemotherapy, lymphoma, cardiac mass, diffuse large b cell lymphoma

## Abstract

Cardiac involvement as the initial presentation of lymphoma is a rare occurrence. The most common type of cardiac lymphoma is diffuse large B-cell lymphoma (DLBCL), which often affects the right atrium. Cardiac lymphoma can either be mediastinal DLBCL invading the heart or primary cardiac lymphoma. We describe the case of an 84-year-old female who presented with an eight-week history of dyspnea. Computed tomography angiography (CTA) of the chest showed a right-sided pleural effusion with collapse of the right middle and lower lobes as well as a large mass-like density within the anterior pericardium, compressing the right atrium and right ventricle and encasing the right coronary artery. A transthoracic echocardiogram (TTE) showed a multilocular hypoechoic mass in the right atrium with invasion into the wall of the right atrium. The patient underwent diagnostic and therapeutic thoracentesis. Pleural fluid cytology revealed diffuse large B-cell lymphoma, with positive stains for CD20, PAX5, CD10, BCL6, and Mum-1. Fluorescence in situ hybridization (FISH) revealed an abnormality of BCL2/18q (16%). A staging positron emission tomography (PET) scan showed a large mediastinal mass involving the right pericardium, focal uptake in the left thyroid lobe, left skull base, and musculature around the proximal left femur. Chemotherapy was initiated with R-mini-CHOP (rituximab, cyclophosphamide, doxorubicin, vincristine, and prednisone). PET scans after three cycles of chemotherapy showed a complete metabolic response with the resolution of previously noted hypermetabolic lesions. The patient completed all six cycles of chemotherapy without issues. The differential diagnosis of a right atrial cardiac mass should include lymphoma. TTE is usually the initial imaging test, and a tissue biopsy is required for a definitive diagnosis. DLBCL is highly aggressive and carries a poor prognosis if untreated. Early diagnosis and treatment with standard chemotherapy are crucial for favorable outcomes.

## Introduction

Cardiac involvement as the initial presentation of lymphoma is a rare occurrence. The most common type of cardiac lymphoma is diffuse large B-cell lymphoma (DLBCL), which often affects the right atrium [1]. Cardiac lymphoma can either be mediastinal DLBCL invading the heart or primary cardiac lymphoma, which is confined to the heart and pericardium [2].

Primary mediastinal DLBCL constitutes 2-4% of all non-Hodgkin's lymphomas. It is a rare and rapidly progressive malignancy. Primary cardiac lymphoma is extremely rare and accounts for 1.3-3% of all primary cardiac tumors [3]. Malignant primary cardiac tumors have an exceedingly poor prognosis without treatment, with a reported 10% survival rate at nine to 12 months without treatment [4].

R-CHOP (rituximab, cyclophosphamide, doxorubicin, vincristine, and prednisone) chemotherapy is the mainstay of treatment. Other regimens include R-pola-CHP (rituximab, polatuzumab vedotin, cyclophosphamide, doxorubicin, and prednisone) [5]. We describe a case of mediastinal DLBCL, which presented as a cardiac mass. We aim to highlight the different ways that DLBCL may present and the importance of early diagnosis and treatment. 

## Case presentation

An 84-year-old Caucasian woman with a past medical history of hypothyroidism, hypertension, and depression presented to the emergency department with shortness of breath. She reported having progressive dyspnea over the last eight weeks, worse with exertion. She also reported a dry cough but denied any fevers, chills, weight change, or chest pain. She did not smoke or drink alcohol. She had no personal history of cancer. She reported a history of colorectal cancer in her mother and maternal aunt. Her physical examination was unremarkable. She was hemodynamically stable. 

The lab workup showed a white blood cell count of 10.7 K/mcL, hemoglobin of 11.3 g/dL, and platelets of 160 K/mcL. D-dimer was elevated to 628 ng/mL DDU. Lactate dehydrogenase (LDH) was 265 units/L. Her kidney function, electrolytes, and hepatic function were within normal limits. High-sensitivity troponin was mildly elevated at 33 ng/L and then 30 ng/L. Her electrocardiogram showed normal sinus rhythm, with T wave inversions in leads V2 through V6, and no changes in the ST segment. 

**Figure 1:**
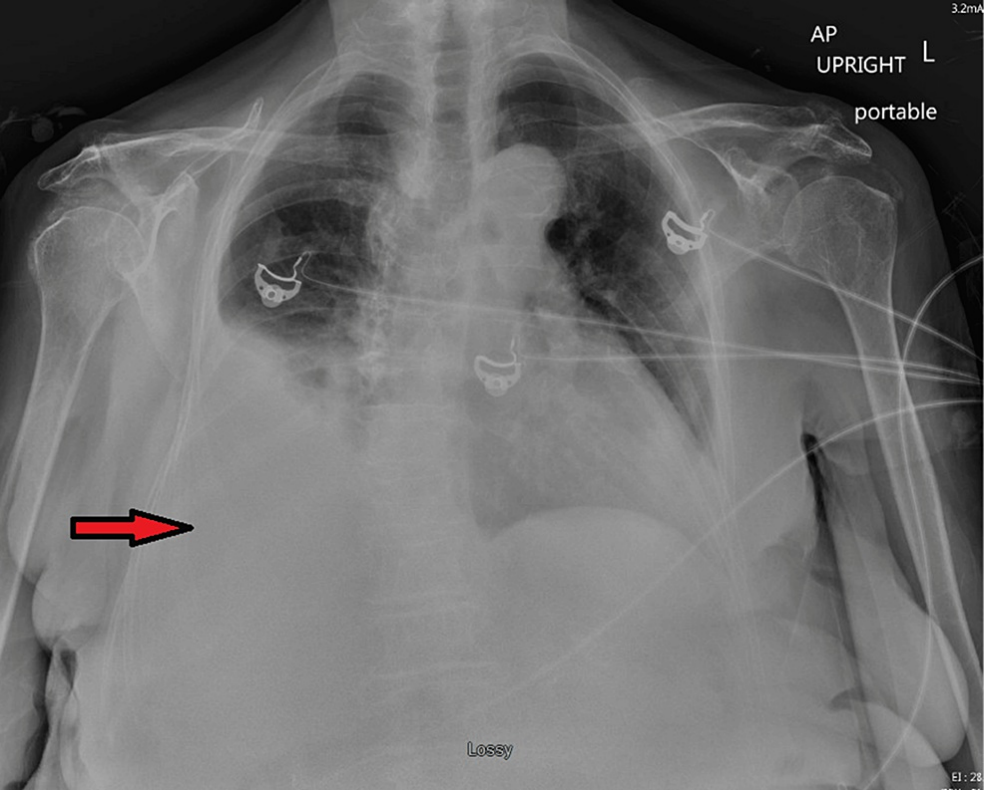
Chest x-ray showing right pleural effusion.

**Figure 2:**
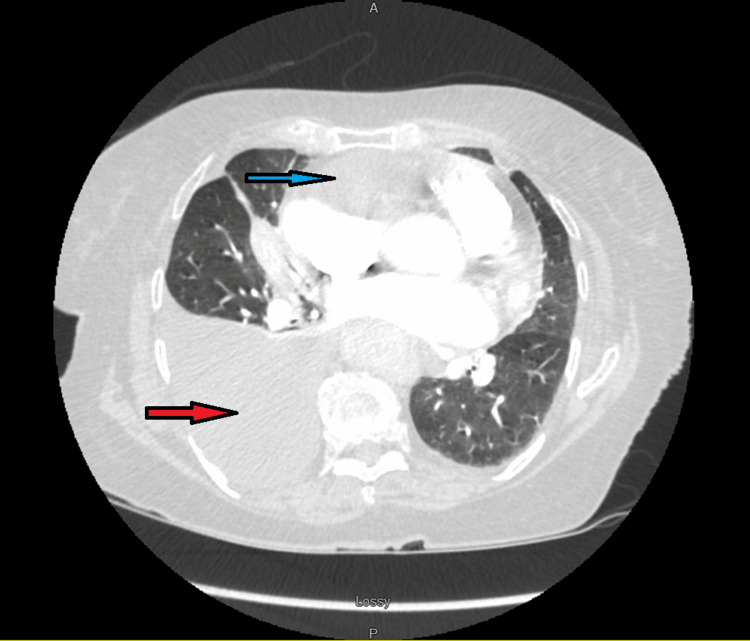
CT angiogram of the chest showing the large mediastinal mass (blue arrow) and right-sided pleural effusion (red arrow).

**Figure 3:**
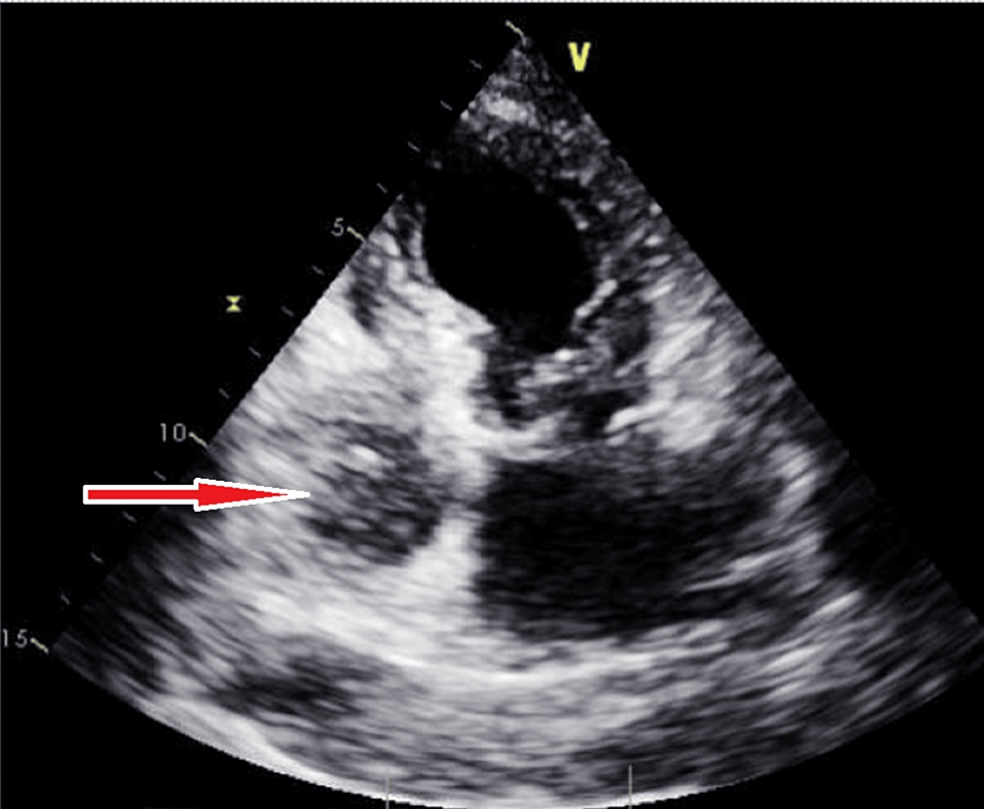
Transthoracic echocardiogram showing the multiloculated hypoechoic mass in the right atrium (arrow).

Chest x-ray showed a new large right-sided pleural effusion (Figure 1). CT angiogram chest showed the right-sided pleural effusion with collapse of the right middle and lower lobes as well as a large mass-like density within the anterior pericardium, measuring 8.5 x 5.2 x 9.4 cm, compressing the right atrium and right ventricle and encasing the right coronary artery. There was also an area of filling defect within the right atrium concerning mass invasion versus thrombus (Figure 2). A transthoracic echocardiogram (TTE) showed a multiloculated hypoechoic mass in the right atrium measuring 7 x 6.7 cm, with some components invading the wall of the right atrium (Figure 3).

The patient subsequently underwent diagnostic and therapeutic right-sided thoracentesis. She had 1.2 L of fluid removed with this and felt symptomatically improved. Pleural fluid studies were consistent with an exudative effusion. Pleural fluid cytology revealed diffuse large B-cell lymphoma and positive stains for CD20, PAX5, CD10, BCL6, and Mum-1. Biopsy of the mass was deferred due to conclusive results from pleural fluid cytology. Fluorescence in situ hybridization (FISH) revealed abnormality of BCL2/18q (16%), negative for BCL6 rearrangement, MYC rearrangement, MYC/IGH rearrangement, and IGH/BCL2 rearrangement. 

**Figure 4:**
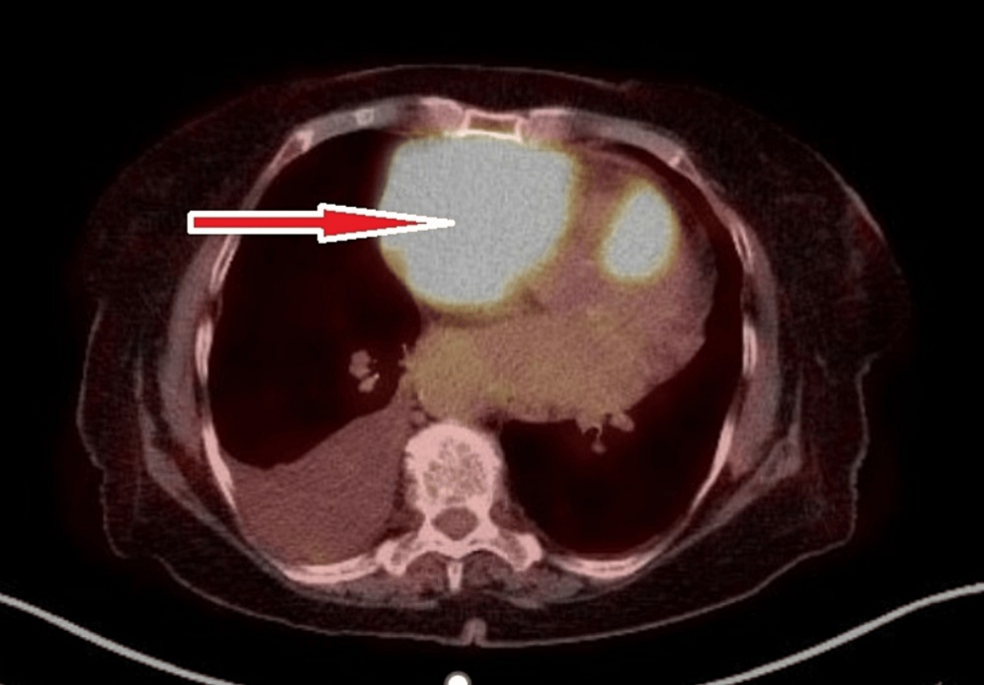
PET scan showing hypermetabolic activity in the anterior mediastinum and pericardium (arrow). PET: positron emission tomography.

A staging positron emission tomography (PET) scan showed a large mediastinal mass involving the right pericardium, focal uptake in the left thyroid lobe, left skull base, and musculature around the proximal left femur (Figure 4). The patient was diagnosed with stage IV diffuse large B-cell lymphoma. Cardiothoracic surgery input was sought for the intracardiac mass; it was felt to be a direct tumor extension inside of the thrombus and the decision was made to not anti-coagulate. She tested negative for HIV and hepatitis B. She was started on allopurinol for tumor lysis syndrome prophylaxis. 

**Figure 5:**
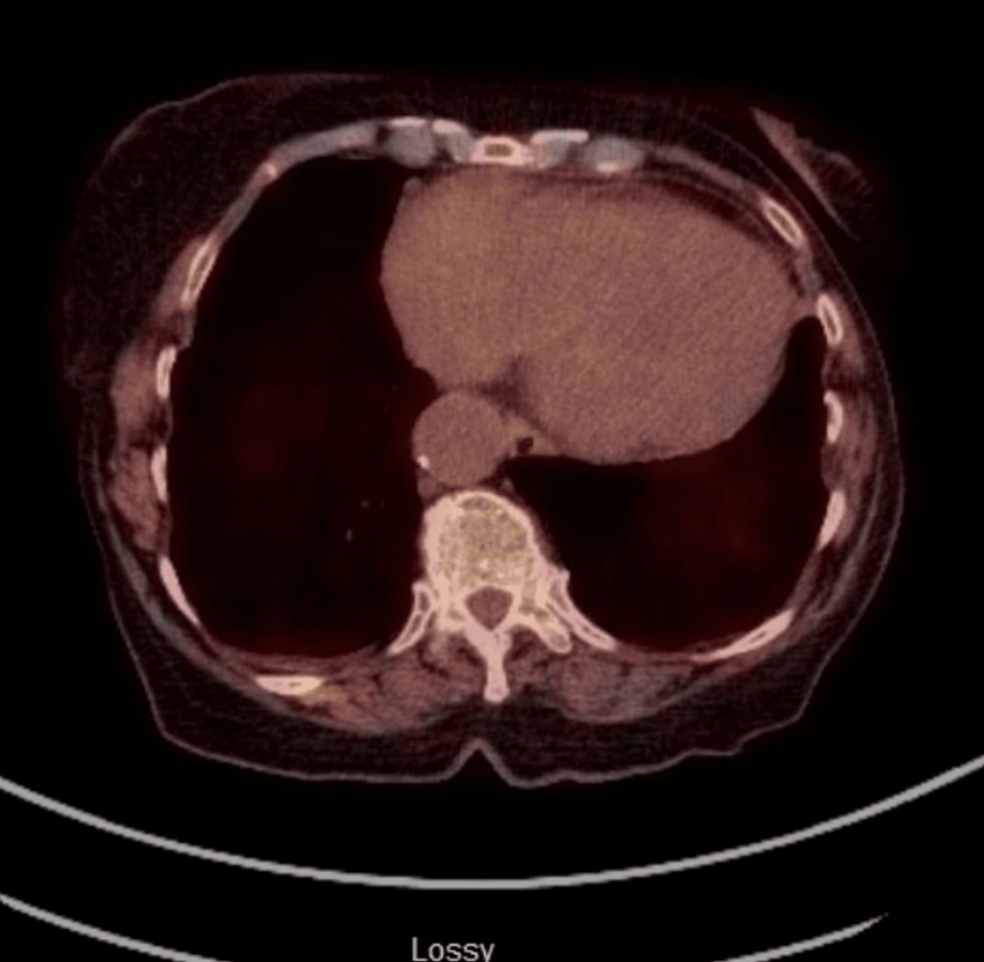
Post-treatment PET scan showing resolution of the hypermetabolic area in the mediastinum. PET: positron emission tomography.

Due to the significant extent of the disease, the decision was made to initiate inpatient chemotherapy. The patient received her first cycle of rituximab, cyclophosphamide, doxorubicin, vincristine, and prednisone (R-mini-CHOP, chosen over traditional R-CHOP due to the patient's advanced age). The patient tolerated this well and was discharged home in stable condition with close oncology follow-up. PET scan after three cycles of chemotherapy showed a complete metabolic response to chemotherapy with a resolution of previously noted hypermetabolic lesions within the neck, thyroid, pericardium, mediastinum, right atrium, and musculature around the left proximal femur (Figure 5).

**Figure 6:**
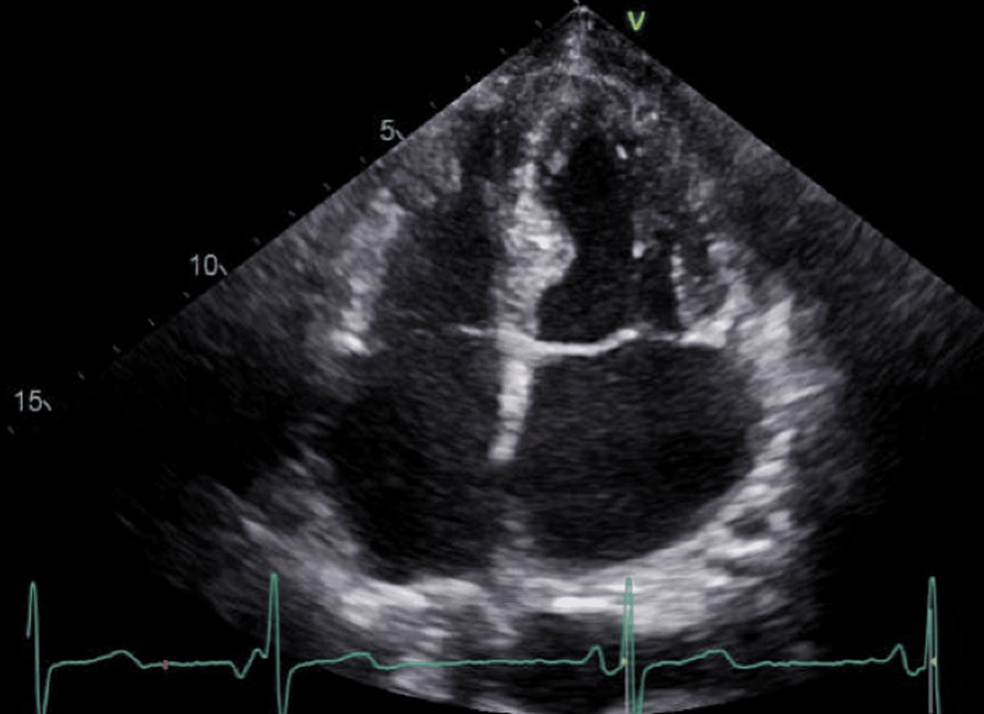
Post-treatment TTE showing resolution of previously noted right atrial mass. TTE: transthoracic echocardiogram.

The patient subsequently completed six cycles of chemotherapy with R-mini-CHOP without complications. A PET scan was repeated after six cycles and showed no recurrence of the disease. TTE post-treatment showed resolution of the previously noted right atrial mass (Figure 6). 

## Discussion

Mediastinal DLBCL with cardiac involvement or primary cardiac lymphoma may present with non-specific features like dyspnea, arrhythmias, or pericardial effusion [2]. On occasion, these cardiac tumors may present as superior vena cava syndrome [6].

The differential diagnosis of a cardiac mass includes benign primary cardiac tumors like myxoma, malignant primary cardiac tumors like primary cardiac lymphoma or rhabdomyosarcoma or angiosarcoma, direct invasion of mediastinal tumors like mediastinal DLBCL, and secondary cardiac tumors, i.e., metastases [3]. Since primary cardiac tumors are so rare, patients with associated mediastinal lymphadenopathy should be evaluated for direct extension of the mediastinal lymphoma [2]. Secondary cardiac tumors are commonly pericardial metastases of primary breast or lung carcinomas, melanoma, or myocardial metastases originating from hematologic or solid tumors [7]. An additional important consideration in a patient with a detected cardiac mass and known systemic malignancy is differentiating metastatic disease from thrombus [8].

TTE is usually the initial imaging test. However, it may underestimate the size of the mass or miss it altogether. TTE is more sensitive for detecting cardiac masses [2]. The diagnosis of cardiac tumors is challenging and often requires multiple imaging techniques [6]. Cardiac MRI has previously been evaluated in diagnostic accuracy for differentiating benign versus malignant cardiac masses. In a study by Mousavi et al., blinded readers correctly diagnosed 89%-94% of cases as benign vs malignant with a 95% agreement rate [9]. PET is indicated for staging and prognostication. Tissue biopsy currently remains the gold standard [2]. In our case, we obtained the diagnosis from pleural fluid cytology instead. 

When a new cardiac mass is identified, the tumor location could be suggestive of the primary etiology. Myxomas are commonly detected in the left atrium [7]. Angiosarcomas, the most frequently occurring malignant primary cardiac tumor, tend to originate from the right atrial wall and extend to the pericardium [10]. Cardiac lymphomas notably may be found in any chamber of the heart or the pericardial space, most commonly in the right atrium [1,3]. Our patient had a pericardial mass with extension into the right atrium, suggestive of lymphoma or angiosarcoma. 

It can be difficult to differentiate mediastinal DLBCL with cardiac involvement from primary cardiac lymphoma, as both present similarly and commonly involve the right side of the heart. CT and cardiac MRI are likely the best methods to differentiate these [3]. The prognosis of mediastinal DLBCL with cardiac involvement is worse than primary cardiac lymphoma. This is in part due to delayed diagnosis, which in turn might be due to a low index of suspicion and rapid growth of the cancer [2,11].

Chemotherapy with R-CHOP has shown good response rates in patients with cardiac DLBCL. In one case series, the overall response rate of primary cardiac lymphoma to this chemotherapy was 79%, and the median overall survival was 30 months [12]. Other options include R-pola-CHP, which had a significantly higher percentage of patients surviving without progression at two years when compared with R-CHOP [5]. 

Though early initiation of chemotherapy is essential, rare fatal events may occur during initiation, including fatal arrhythmia, massive pulmonary embolism, myocardial injury, and a possible theoretical risk of cardiac rupture [11]. This risk of rupture is especially concerning with diffuse large B-cell lymphoma, though reportedly the risk may be mitigated with guidance from cardiac imaging [13].

Surgical resection can be considered in the early stages of the disease in addition to chemotherapy [6,14]. Patients presenting with superior vena cava syndrome and hemodynamic compromise require debulking surgery [6]. In cases of severe right ventricular outflow obstruction, surgery may be palliative to improve blood flow to the lungs [14]. Radiation may be used for patients with progressive disease despite chemotherapy [11]. 

Though cardiac DLBCL has a poor prognosis if untreated, treatment with chemotherapy can be curative, such as in our patient, who had a complete response demonstrated on post-treatment PET scan and TTE. Different factors are thought to impact the prognosis as well, such as bone marrow involvement, extra-nodal involvement, left ventricular involvement, and immunocompromise [11,15].

## Conclusions

Cardiac DLBCL may present with subtle features, and multi-modal imaging techniques are usually required for diagnosis. Clinicians should consider lymphoma in the differentials of a cardiac mass. Mediastinal DLBCL with cardiac involvement should be differentiated from primary cardiac lymphoma, as the prognosis is different. Given that DLBCL is highly aggressive and rapidly growing, early diagnosis and treatment with standard chemotherapy are crucial for favorable outcomes. 
